# Radial Extracorporeal Shock Wave Therapy Enhances the Proliferation and Differentiation of Neural Stem Cells by Notch, PI3K/AKT, and Wnt/β-catenin Signaling

**DOI:** 10.1038/s41598-017-15662-5

**Published:** 2017-11-10

**Authors:** Jing Zhang, Nan Kang, Xiaotong Yu, Yuewen Ma, Xining Pang

**Affiliations:** 1 Department of Rehabilitation Medicine, The First Hospital of China Medical University, Shenyang, Liaoning PR China; 2 Institute of Meta-Synthesis Medicine, Beijing, China; 3 Department of Stem Cells and Regenerative Medicine, Key Laboratory of Cell Biology, Ministry of Public Health and Key Laboratory of Medical Cell Biology, Ministry of Education, China Medical University, Shenyang, Liaoning PR China

## Abstract

Neural stem cell (NSC) proliferation and differentiation play a pivotal role in the repair of brain function in central nervous system (CNS) diseases. Radial extracorporeal shock wave therapy (rESWT) is a non-invasive and innovative treatment for many conditions, yet little is known about the effects of this treatment on NSCs. Mouse NSCs (NE-4C) were exposed to rESWT with 1.0, 1.5, 2.0, 2.5, 3.0, and 3.5 bar (500 impulses, and 2 Hz) *in vitro*. Cell viability test results indicated that rESWT, at a dose of 2.5 bar, 500 impulses, and 2 Hz, increased NE-4C viability within 72 h, and that the PI3K/AKT pathway was involved in its mechanisms. Exposure to rESWT also affected proliferation and differentiation of NE-4C after 8 weeks, which may be associated with Wnt/β-catenin and Notch pathways. This assessment is corroborated by the ability of inhibitors of Wnt/β-catenin [Dickkopf-1 (Dkk-1)] and the Notch pathway (DAPT) to weaken proliferation and differentiation of NSCs. In summary, a proper dose of rESWT enhanced NSCs augment via the PI3K/AKT pathway initially. Also, Wnt/β-catenin and the Notch pathway play important roles in regulation of the long-term efficacy of rESWT. This study reveals a novel approach to culture NSCs *in vitro* and support neurogenesis.

## Introduction

The loss of neurons is a common basis of many neurological diseases affecting the brain and spinal cord and is responsible for an extremely high burden on patients’ lives. Thus, neurogenesis is critical for the treatment of conditions such as multiple sclerosis, ischemic stroke, and neurodegenerative disorders^1^. Neural stem cells (NSCs), which have a proliferation capacity of self-renewal and generation of both neurons and glia^2^, play a key role in endogenous restoration in the mammalian central nervous system (CNS)^3^. Although neuronal regeneration in the brain declines with age^4^, NSCs survive throughout life in a few distinct neurogenic zones, such as the subgranular zone of the hippocampal dentate gyrus and the subventricular zone (SVZ) of the lateral ventricle^5^. Endogenous NSCs in these regions are activated after injury^6^ and generate mature neurons through a complex sequence of developmental steps, including self-renewal, differentiation, migration, targeting, and synaptic integration^5,7^. However, the quantity of activated endogenous NSCs is not sufficient to completely repair nerve injury^3^. NSCs can be isolated and expanded *in vitro* ^8^, and recent data show that NSCs can serve as cell replacement therapies for neurological disorders^9^. For example, transplantation of NSCs can reduce infarct volume and neurological deficits after ischemia stroke^10^. Hence, how to increase the number of NSCs cultured *in vitro* has become a major research focus.

Many researchers have studied the characteristics and regulatory mechanisms of NSCs to better culture NSCs. The proliferation and final fate of NSCs depend on the activation of growth factors and specific signaling pathways, such as PI3K (phosphatidylinositol 3-kinase)/AKT^11^, Wnt/β-catenin^12^, and Notch signaling^13^. The PI3K/AKT canonical pathway is involved in the self-renewal and survival of NSCs^14^. Wnt signaling is instrumental in the differentiation of many adult stem cells^15^. In the central nervous system (CNS), Wnt/β-catenin signaling is also crucial for instructing cell fate choices and regulating neuronal differentiation process^16,17^. Notch signaling is a novel pathway that influences many aspects of NSCs^18^; for examples, Notch signaling is important in the proliferation and fate of NSCs by maintaining the self-renewable state of NSCs, both *in vivo* and *in vitro* ^19^, and plays an essential role in differentiation to neurons^20^. Notch1, a member of Notch pathway receptor, and its cognate ligand Jagged1 are expressed in the neurogenic niches^21^, especially in the adult hippocampus^22^, which take part in the development of the CNS^20^.

The use of extracorporeal shock wave therapy (ESWT) began in the urological field for lithotripsy and gradually extended to many musculoskeletal diseases and regenerative medicine disorders^23^. Presently, two types of ESWT are used in clinical medical therapy; they are, focused ESWT (fESWT) and radial ESWT (rESWT)^24^. fESWT significantly augments migration and proliferation in fibroblasts and keratinocytes, which improves the process of healing^25^, and fESWT has also been shown to have potential as a non-invasive and innovative technology for the treatment of nerve damage, such as sciatic injury^26^ and cerebral ischemia^27^. Although corresponding studies about rESWT are few, several have addressed potential mechanisms. Recently, our studies showed that an appropriate rESWT dose delivered to rat brain helped restore neurological function and increased the number and differentiation of NSCs *in vivo* ^28^. In addition, an effect of rESWT on cells has been demonstrated^29^. and different cell types are differentially influenced by the effects of rESWT^30^. In this study, we used NE-4C mouse NSCs to assess the effect by rESWT on proliferation and differentiation and to further explore the possible signaling pathway of PI3K/AKT, Wnt/β-catenin, and Notch signaling *in vitro*.

## Results

### rESWT increased proliferation and differentiation of NE-4C stem cells

**Figure 1:**
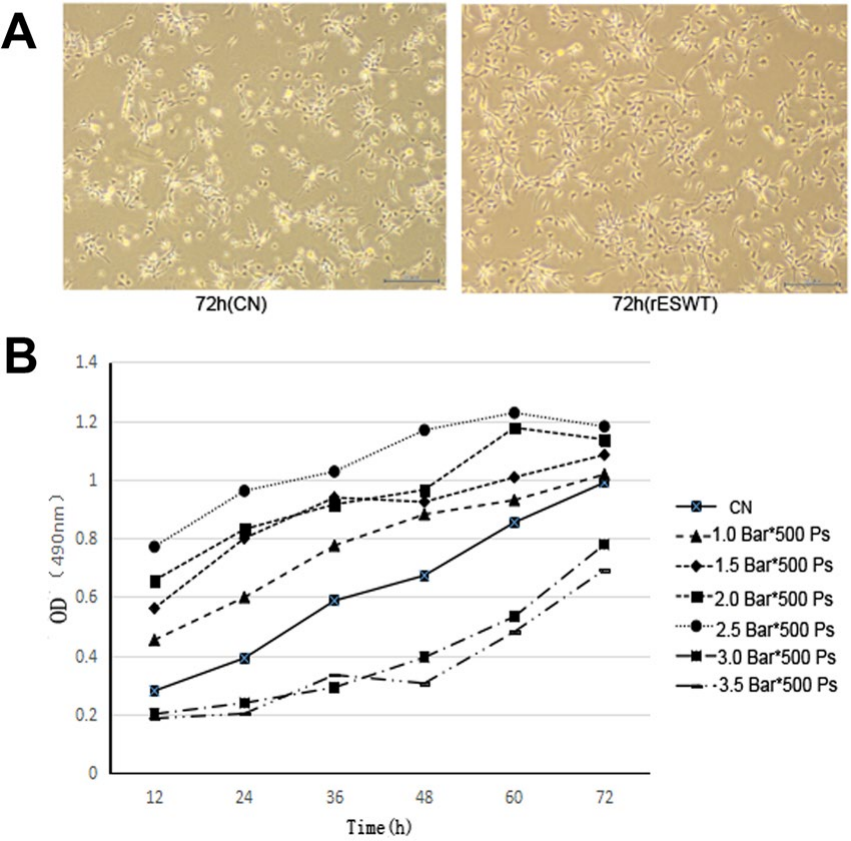
The effect of different doses of rESWT applied on the cultures. The multiplication of NE-4C cells was analyzed using an MTT cell proliferation assay (Promega, Japan). rESWT at a dose of 2.5 bar, 500 impulses, 2 Hz significantly stimulated the number of NE-4C cells at 72 h compared with the control group. Bar: 20 μm. (**A**) Different doses of rESWT ranging from 1.0 to 3.5 bar were used in cultures. The proliferation of NE-4C cells was tested at 12, 24, 36, 48, 60, and 72 h after rESWT. Below the 2.5 bar, 500 impulses, 2 Hz dose, rESWT increased proliferation in a quantity-dependent manner. Over the 2.5 bar, 500 impulses, 2 Hz dose, rESWT had an opposite effect (**B**).

**Figure 2:**
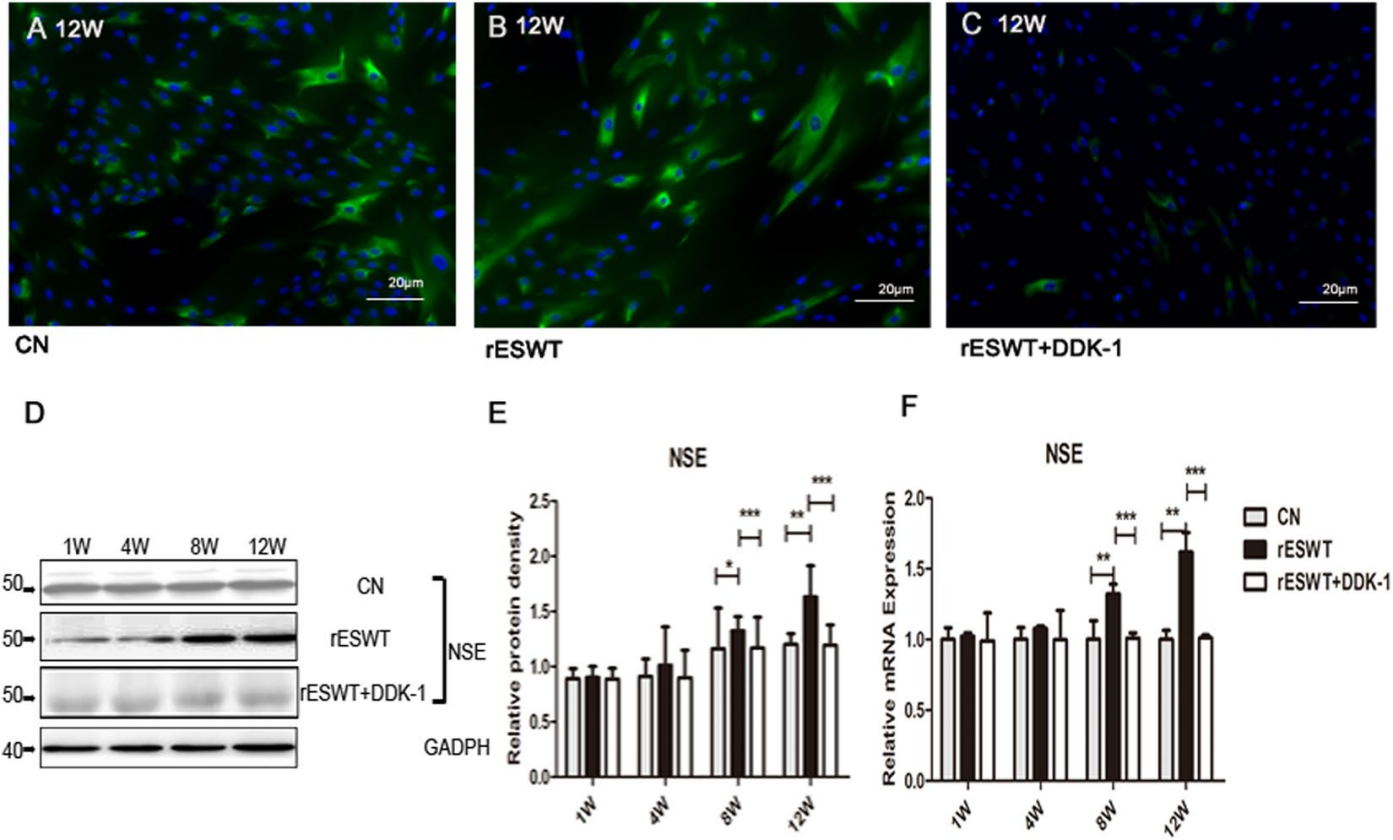
NSE expression. NSE expression was assessed by immunofluorescence, Western blot analysis, and qRT-PCR. Fluorescence signals were analyzed by an Axiovert 200 inverted microscope. Nuclei were stained with DAPI. Bar: 20 μm (**A–C**). The Western blot shows a visible difference in three groups (**D**). Data are reported as the mean ± SD of significant difference from the control group at both week 8 (*P < 0.05) and week 12 (**P < 0.01) and from the Dkk-1 inhibitor group (***P < 0.001) (**E**). NSE mRNA expression was improved at 8 and 12 weeks compared with the control group (**P < 0.01) and Dkk-1 inhibitor group (***P < 0.001) (**F**).

**Figure 3:**
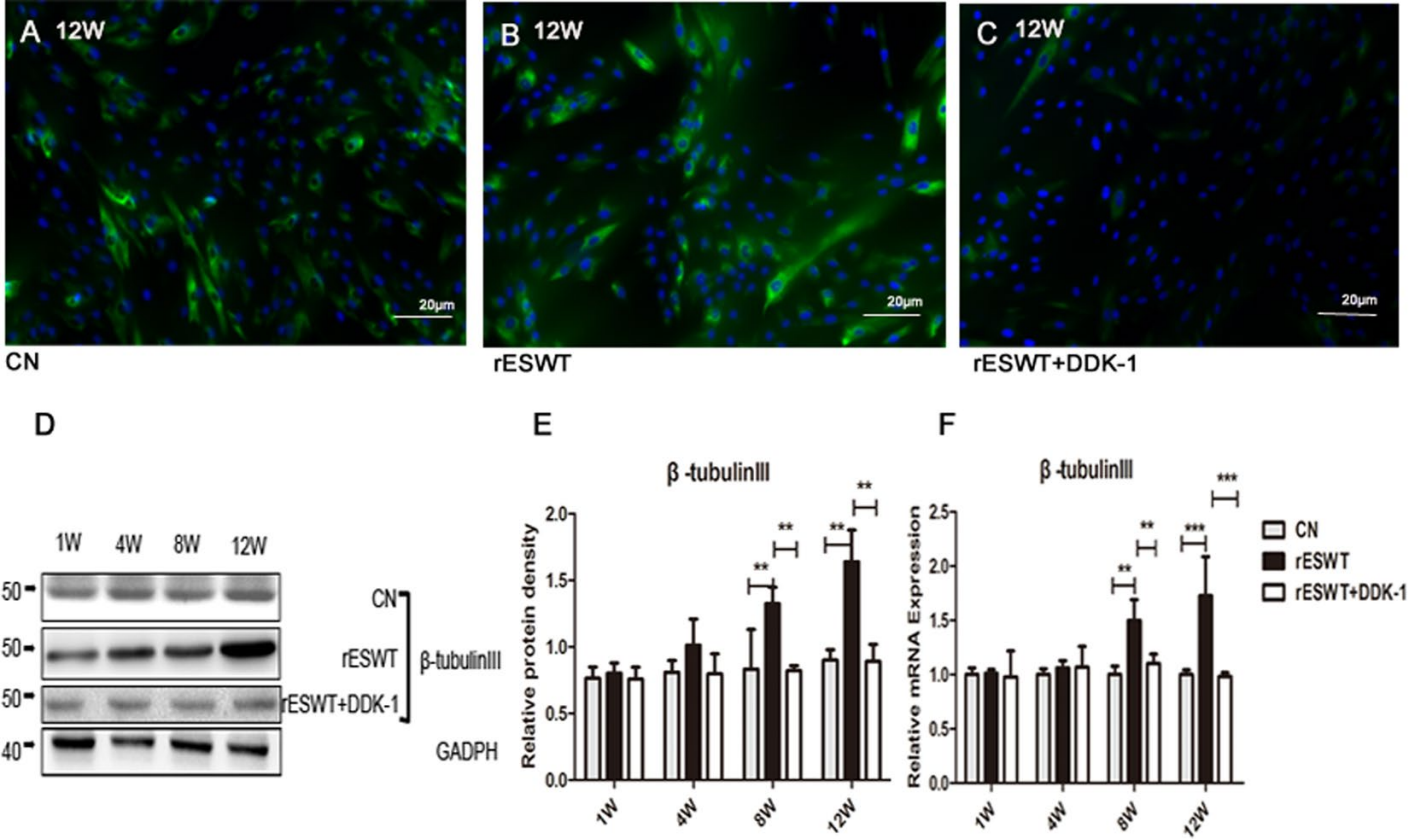
β-tubulin III expression. β-Tubulin III expression was improved at 8 and 12 weeks compared with the control group. The Dkk-1 inhibitor suppressed β-tubulin III expression with regard to both the protein (**A–E**) and mRNA (**F**) levels as determined by immunofluorescence staining (**A–C**), Western blot (**D,E**), and qRT-PCR (**F**) analyses. β-Tubulin III fluorescence is shown in green and nuclei staining is shown in blue. Bar: 20 μm (**p < 0.01, ***P < 0.001).

An rESWT dose of 2.5 bar, 500 impulses, and 2 Hz significantly increased the NE-4C stem cells 72 h after rESWT compared with the control group (Fig. 1A). The rESWT quantity dose-dependently stimulated proliferation below 2.5 bar, 500 impulses, and 2 Hz. Interestingly, when rESWT exceeded the aforementioned dose, an opposite effect was observed (Fig. 1B). An rESWT dosage of 2.5 bar, 500 impulses, and 2 Hz was used in this study. Neuron-specific enolase (NSE) and β-tubulin III expression levels were measured by immunofluorescence, Western blot, and quantitative reverse-phase polymerase chain reaction (qRT-PCR). All cultures revealed positive expression for NSE and β-tubulin III, the marker of neuronal cells, on weeks 8 and 12 after the onset of rESWT (Figs 2 and 3), but no difference was discovered before week 8. The fluorescent marker of the control group was significantly less than that of the experimental group (Figs 2A–C, 3A–C). Results of Western blot and qRT-PCR analyses (Figs 2D–F, 3D–F) are consistent with the immunofluorescence results.

### rESWT increased the protein expression of PI3K and mRNA expression of PI3K and AKT

**Figure 4:**
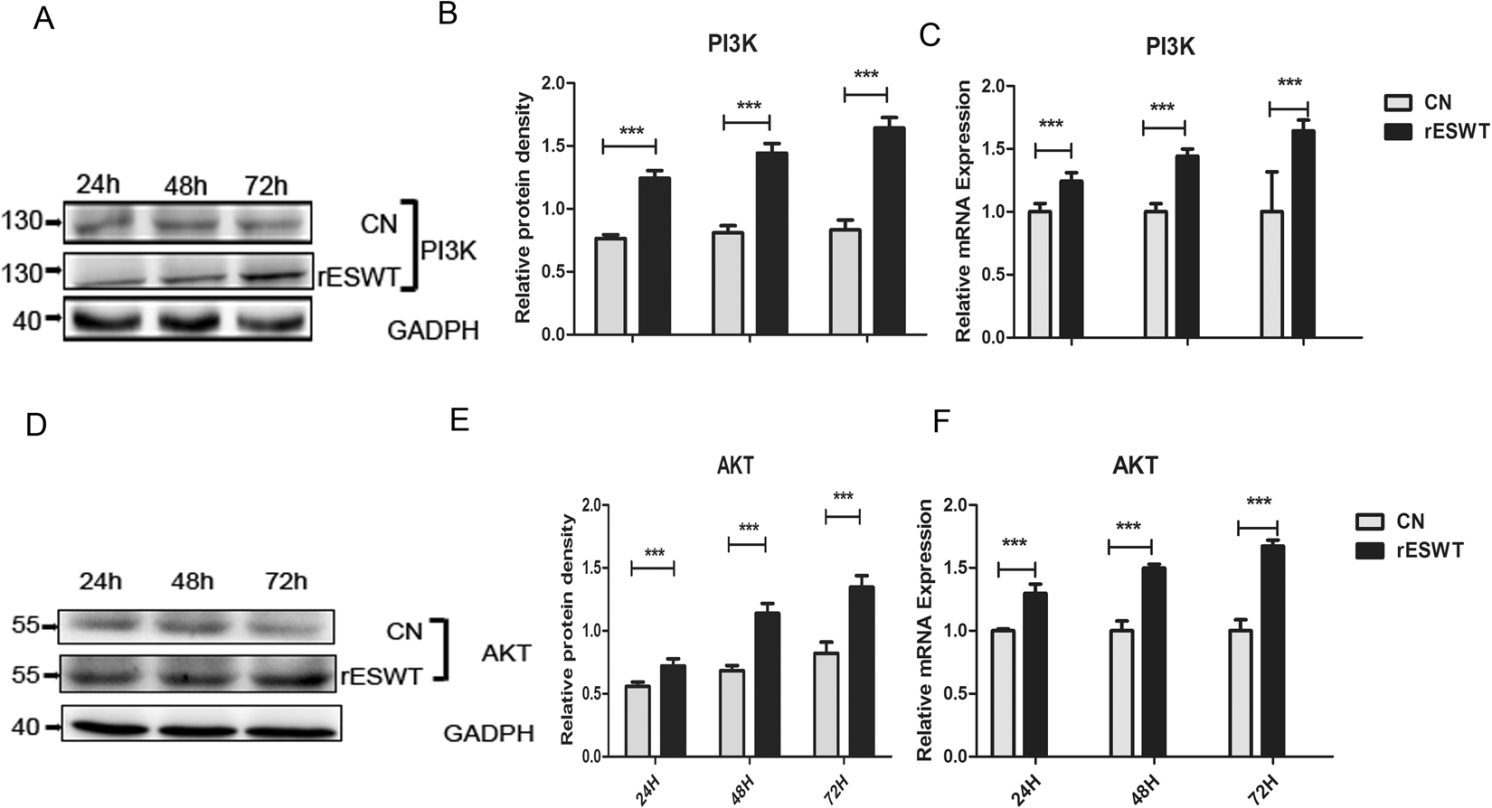
Western blot and Real-time PCR findings of PI3K and AKT. The Western blot results demonstrate the enhanced expression of PI3K and AKT at 24, 48, and 72 h after rESWT in two independent experiments (***P < 0.001) (in **A**,**B**,**D**,**E**). PI3K and AKT mRNA expression, as measured with qRT-PCR, are also increased at 24, 48, and 72 h using rESWT (***P < 0.001) (in **C,F**).

The expression of PI3K and AKT was measured by Western blot and qRT-PCR. Compared with the control group, rESWT distinctly (P < 0.001) promoted PI3K and AKT expression at 24, 48, and 72 h (Fig. 4A,B,D,E). The mRNA expression levels of PI3K and AKT were also increased (P < 0.001) after rESWT (Fig. 4C,F), which is consistent with the time of stimulating the cell proliferation.

### The Wnt/β-catenin signaling pathway was induced by rESWT

**Figure 5:**
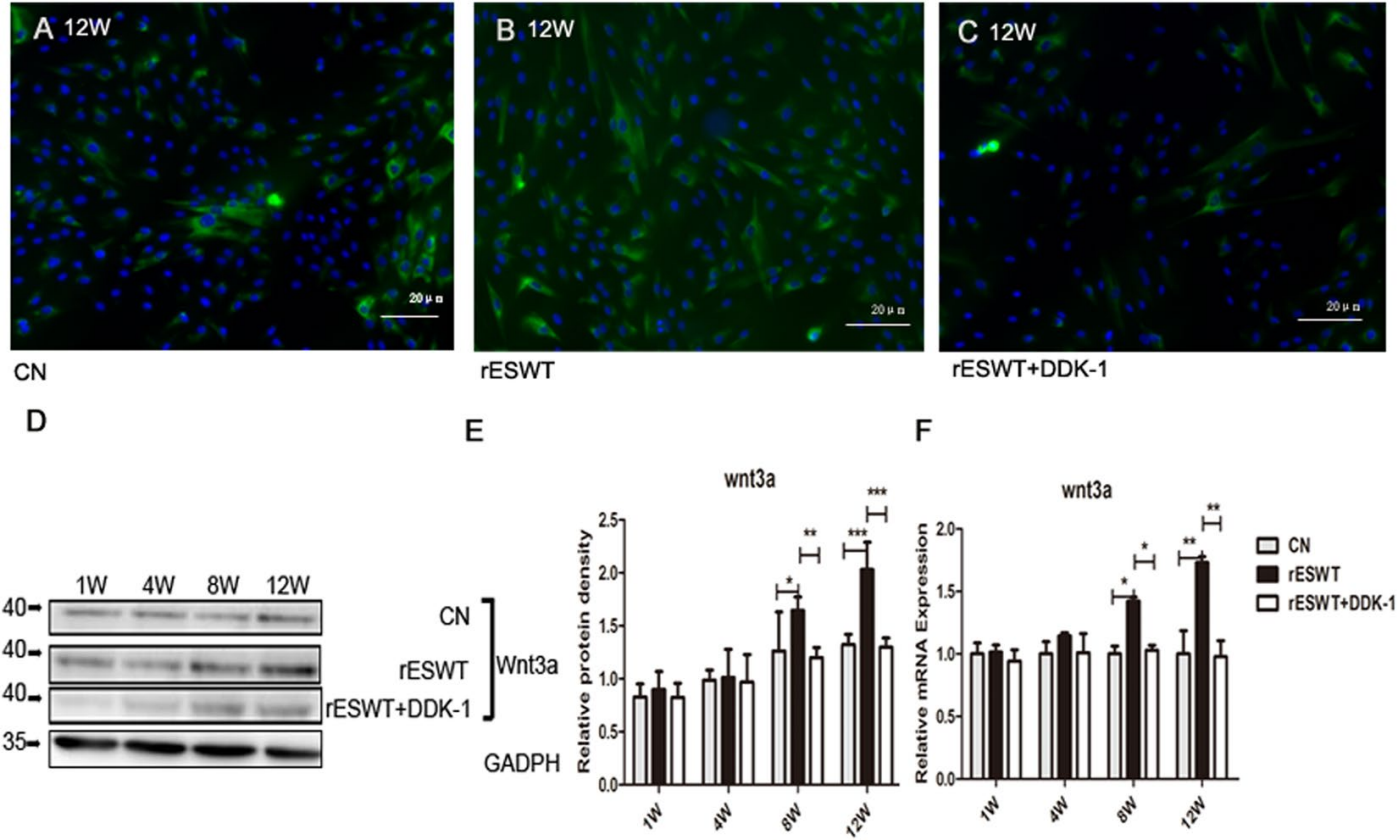
Wnt3a expression. Wnt3a expression was determined at 1, 4, 8, and 12 weeks. Immunofluorescence microscopic findings of Wnt3a (green color) show that the expression increased compared with the control group and decreased in the Dkk-1 inhibitor group (**A–C**). Western blot findings demonstrate that the prominent improvement in expression was revealed only at 8 and 12 weeks relative to the control group (**D,E**). The same results are seen for the mRNA expression (**F**). (*P < 0.05, **P < 0.01, ***P < 0.001). The data are presented as the mean ± SD.

**Figure 6:**
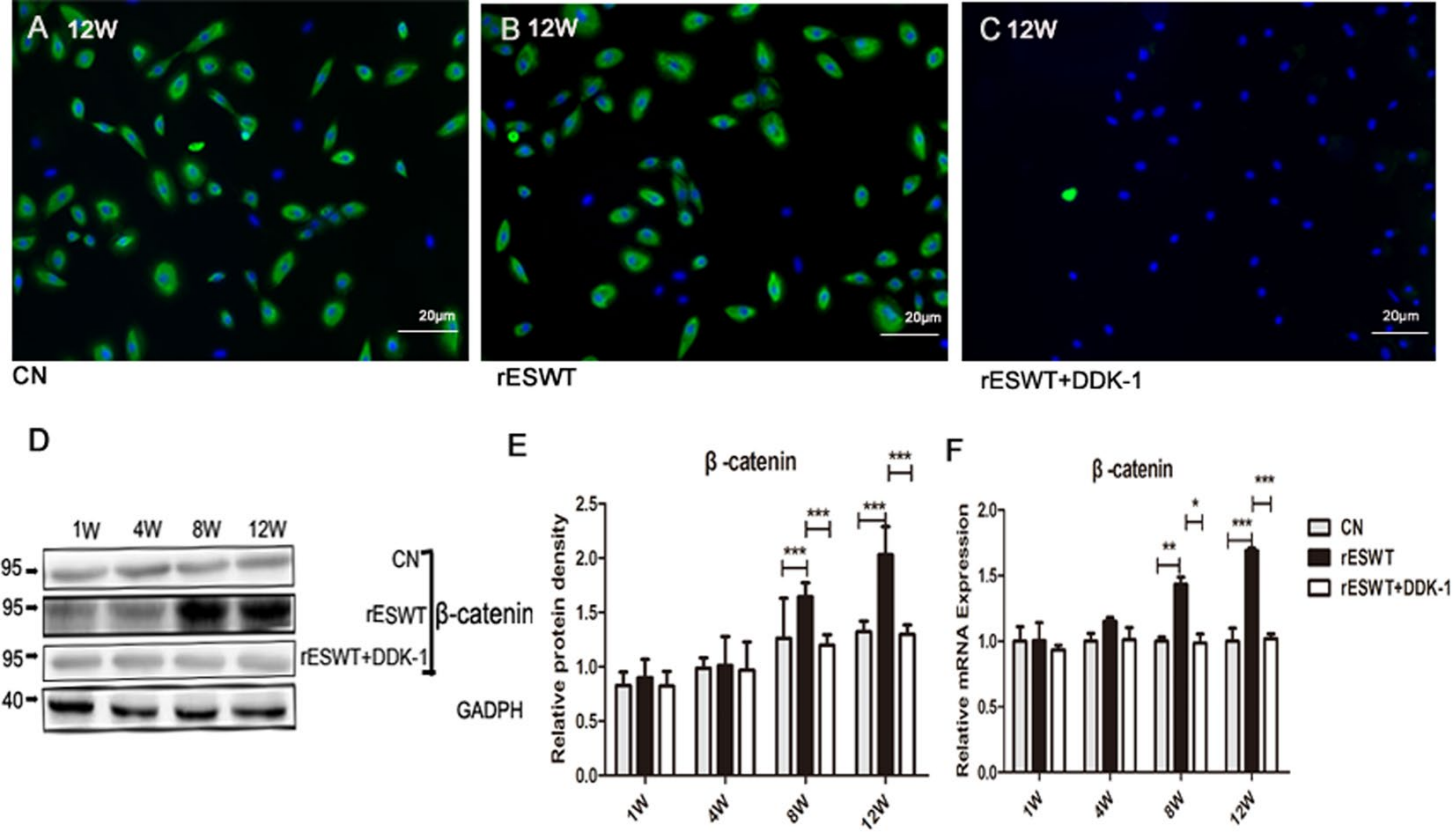
β-catenin expression. β-catenin (green color) immunofluorescence findings (**A–C)**, and Western blot findings (**D,E**) demonstrate the augmented expression at weeks 8 and 12 compared with the control group, and reduced expression induced by the Dkk-1 inhibitor (***P < 0.001). β-catenin mRNA expression was also improved relative to control group at 8 weeks (**P < 0.01) and 12 weeks (***P < 0.001) and reduced in the Dkk-1 inhibitor group at 8 weeks (*P < 0.05) and 12 weeks (***P < 0.001) (**F**). Data are reported as the mean ± SD.

**Figure 7:**
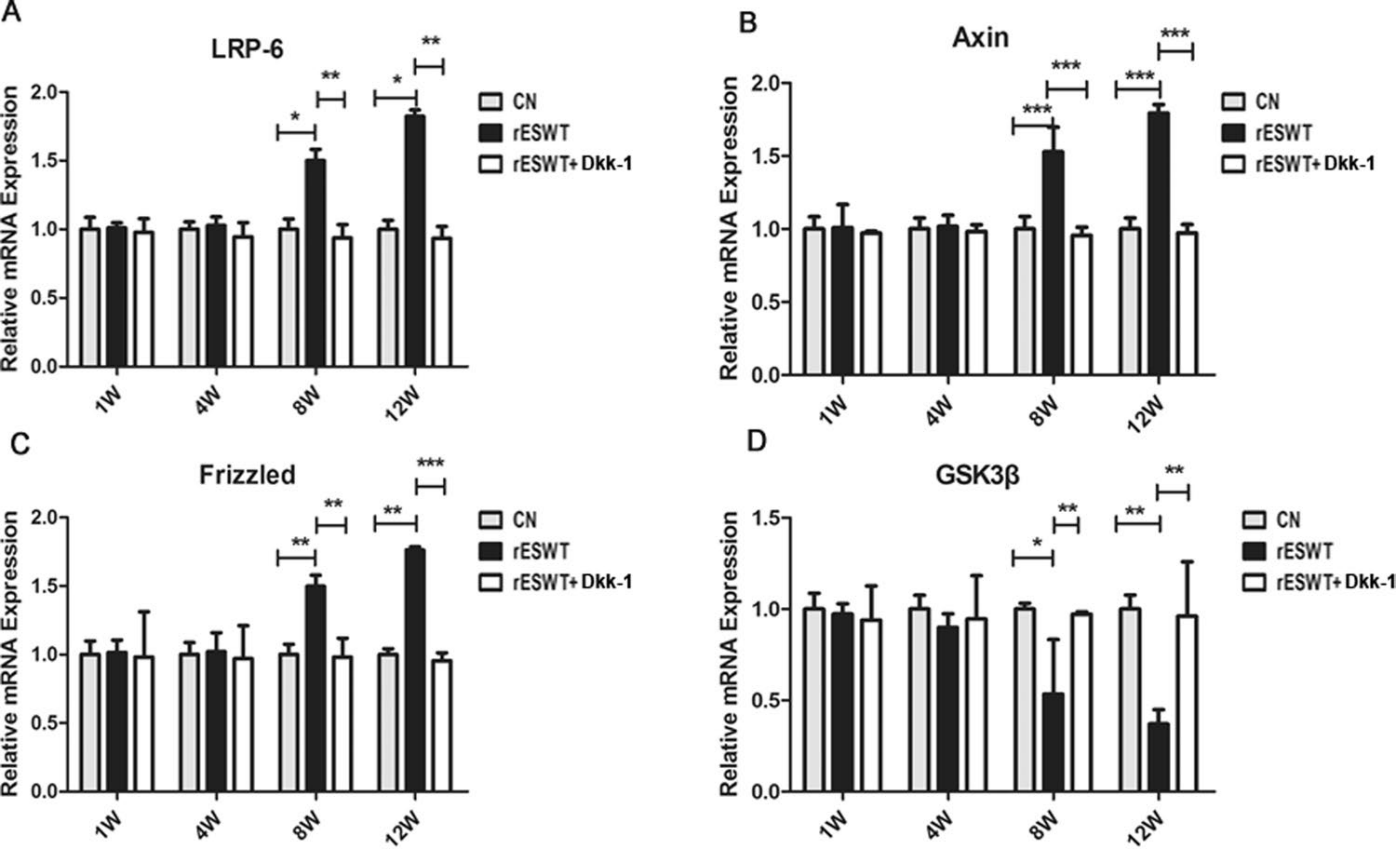
LRP-6, Axin, Frizzled, and GSK3β expression. qRT-PCR was used to test the mRNA expression of LRP-6, Axin, Frizzled, and GSK3β. Results revealed distinct differences between three groups on weeks 8 and 12. LRP-6, Axin, and Frizzled mRNA expression increased after rESWT and was suppressed by the Dkk-1 inhibitor (**A–C**). GSK3β expression was opposite to that seen for LRP-6, Axin, and Frizzled mRNA (**D**). (*P < 0.05, **P < 0.01, ***P < 0.001).

The Wnt3a and β-catenin response was induced on week 8 and maintained steadily to week 12. rESWT unregulated the Wnt3a and β-catenin protein expression and the mRNA expression detected by immunofluorescence, Western blot, and qRT-PCR (Figs 5 and 6). The mRNA expression of other key points of the Wnt/β-catenin pathway were also altered; for examples, mRNA expression of low-density lipoprotein receptor-related protein 6 (LRP-6), Axin, and Frizzled increased, whereas that of the glycogen synthase kinase 3β (GSK3β) decreased (Fig. 7). Dkk-1, the inhibitor of Wnt/β-catenin pathway, apparently suppressed the expression of Wnt3a and β-catenin, and the other key point of Wnt/β-catenin pathway, as well as NSE and β-tubulin III expression after rESWT (Figs 2, 3, 5, 6, and 7).

### rESWT unregulated Notch signaling pathway in agreement with nestin and NSE expression

**Figure 8:**
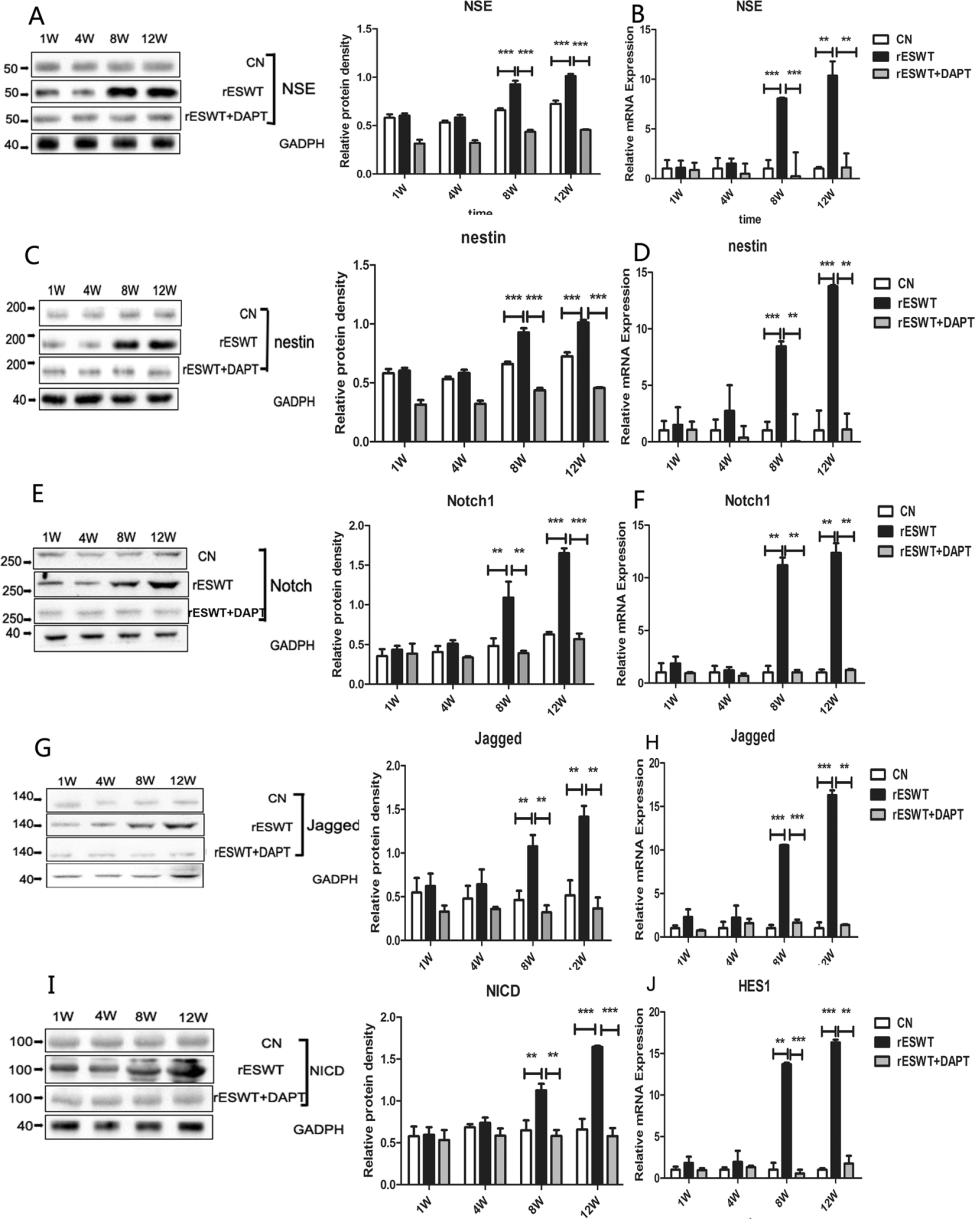
Notch1 signaling, nestin, and NSE expression after rESWT. Notch1, NICD, and Jagged1 expression was enhanced after rESWT on weeks 8 and 12 (**P < 0.01, ***P < 0.001) and suppressed by DAPT (**E,G,I**). mRNA expression for Notch1, Jagged1, and Hes1 was augmented compared with the control group and DAPT group (**P < 0.01, ***P < 0.001) (**F,H,J**). Notch1 signaling was upregulated and expression of NSE, nestin, and mRNA was increased and was inhibited by DAPT on weeks 8 and 12 (***P < 0.001, **P < 0.01) (**A–D**).

Notch1, Jagged1, and Hes1 expression augmented gradually on week 8 and week 12 after rESWT shown by Western blot and qRT-PCR (Fig. 8E–J). This increasing trend was not only consistent with the expression of NSE (Fig. 8A,B), but also in accordance with NSC marker nestin expression (Fig. 8C,D). In addition, the γ-secretase inhibitor DAPT suppressed the Notch1 signaling but also reduced the expression of NSE and nestin.

## Discussion

The results of the current study demonstrate that rESWT promoted the proliferation and differentiation of mouse NSCs *in vitro*. As the second generation of fESWT, with waves dispersing eccentrically from the applicator tip^31^, rESWT is a breakthrough in clinical medicine because of its better security than fESWT, its simplicity of use, and especially for its excellent therapeutic results^32^; for example, rESWT simplifies application without anesthesia or image-guided location and lessens risks by reflecting the pathology zone^32^. Also, rESWT has many transmitters with various penetration depths, which might allow rESWT to satisfy various clinical needs^33^. In recent years, fESWT has aroused the interest of more and more cell researchers who have reported that fESWT enhances the differentiation of human tendon-derived stem cells^29^ and stemness of rat and human adipose-derived stem cells^34^. However, few studies have yet explored the effects of rESWT on NSCs. Due to the complicated culture and differentiation of NSCs *in vitro* ^8^ and potential usefulness of rESWT, exploration of rESWT is imperative. Consequently, this study investigated the effect of rESWT on NSCs *in vitro*.

An appropriate rESWT dose had a powerful effect on NE-4C proliferation within 72 h. The rESWT quantity dependently increased the self-renewal of NSCs within a certain range (below 2.5 bar, 500 impulses, and 2 Hz), which is similar to the report that exposure to rESWT resulted in a number of rESWT-dependent alterations after 48 and 72 h on human fetal foreskin fibroblasts and that the effect may attribute to the mechanical stimulation of rESWT^30^. However, higher energy rESWT induced cell damage and apoptosis. This result is also found using fESWT, which may be related to increasing severity of the shock wave conditions that can increase apoptosis^35^.

NSCs survival *in vitro* involves the activation of the PI3K/AKT pathway^36^. PI3K is a family of enzymes that phosphorylate the 3’-OH of the inositol ring of phosphatidylinositol^37^. The action of PI3K leads to phosphorylation of AKT to p-AKT. p-AKT could then induce the phosphorylation of the transcription factor FOXO3a, leading to upregulation of Cyclin D1, which increases the number of NSCs^38,39^. Moreover, past studies have shown the inhibition of PI3K/AKT pathway reduces cell divison^36^. These studies emphasize that the PI3K/AKT pathway is vital to proliferation of NSCs. Consistent with previous studies, our data indicate that the PI3K/AKT pathway was activated when NE-4C cells number increased. Compared with the control group, rESWT promoted the proliferation of NSCs, while greatly upregulating PI3K and AKT expression within 72 h *in vitro*. These results demonstrate that rESWT augmentation of the number of NSCs may be associated with the PI3K/AKT pathway in the early period.

In addition to proliferation, differentiation of NSCs also has a certain role in neurogenesis. In our research, rESWT can not only affect the augment of NSCs, but also boost the regulation to neurons. The neurons markers NSE and β-tubulin III, were, measured to characterize neuronal differentiation. NSE and β-tubulin III expressions were higher on week 8 after rESWT than those in the control group, which indicates that rESWT enhanced the differentiation of NE-4C into neuron-like cells. In fewer than 8 weeks, however, the expression of NSE and β-tubulin III was not increased, which may be due to a long-term need for formation of neurons. With respect to neural development, Wnt/β-catenin signaling is critically required for NSCs differentiation^40^. Wnt3a, a member of Wnt family, has been shown to promote NSCs maturity^41^. Upon Wnt3a binding to the Frizzled receptor, adenomatous polyposis coli (APC) is dissociated and Axin is removed from the Axin/GSK3β/APC complex. Axin’s interaction with the Wnt co-receptor LRP-6 causes APC to dissociate from Axin. As a consequence, β-catenin is stabilized, translocates to the nucleus, and interacts with transcription factors, which in turn induces gene expression and causes cells to differentiate^41–43^. Previous studies showed that the β-catenin activation in NSCs induces improvement of neuronal number in the adult forebrain^44^. To explore whether the powerful effect of rESWT on NSCs differentiation is related to the Wnt/β-catenin pathway, Wnt3a, β-catenin, and other key points of Wnt/β-catenin pathway, such as LRP-6, Axin, Frizzled, and GSK3β expression, were detected. Our investigation indicates that rESWT enhanced cell differentiation on week 8 with the activation of the Wnt/β-catenin signal. With the application of rESWT, Wnt3a and β-catenin increased significantly after 8 weeks. Moreover, inhibition of Wnt/β-catenin signal pathway with Dkk-1^45^ significantly reduced the neuronal maturation of NSCs as evidenced by decreased NSE and β-tubulin III expression. These findings provide further evidence that the rESWT treatment modulates NSCs differentiation via the Wnt/β-catenin signal. Although there are a large number of studies regarding the Wnt/β-catenin pathway, our work is the first that reveals a new strong role of rESWT in NSCs differentiation *in vitro* that is indicated by the upregulated Wnt/β-catenin pathway.

Recent research of regulating maintenance and neuronal development of NSCs has identified additional pathways including the PI3K/AKT pathway and Wnt/β-catenin signal, Notch pathway^46^. In our study, Notch1, Jagged1, and Hes1 expression were augmented on week 8 and week 12 after rESWT compared with the control group. Notch1 plays a critical role in the development of the CNS^47^, and higher levels of *in vitro* neuronal differentiation in human NSCs have been observed^48^. Notch1, when activated by transmembrane ligands Jagged1, is cleaved by the Presenilin γ-secretase complex, which liberates Notch1 intracellular domain (NICD) fragments. Then, NICD is released and translocated to the nucleus where it induces target gene expression (in particular, Hes1)^49^. The key point of an increase in Notch pathway expression, which is consistent with the expression of the neuron marker NSE, is that Notch1 signaling can also be considered to be a potent modulator of NSCs maturation and the Wnt/β-catenin signal, as reported in previous studies that NSCs differentiation is driven by increased Notch pathway^50^. NSE expression stimulated by rESWT was significantly suppressed by using the Notch 1 signaling inhibitor DAPT. All these data suggest that the Notch1 signal is involved in inducing the differentiation of NSCs by rESWT. Similar to the Notch1 signal, expression of the NSCs marker nestin was higher in the rESWT group than the control group on weeks 8 and 12, and was reduced by DAPT. In light of the known effect of Notch1 signaling maintaining t NSCs self-renewal^51^, one of the mechanisms of rESWT-induced NSC proliferation at a later period *in vitro* is Notch1 signaling.

The results of the present study illustrate for the first time that rESWT improved the proliferation and differentiation of NSCs *in vitro*. This effect was likely caused, at least in part, by an upregulated PI3K/AKT pathway, Wnt/β-catenin signaling, and Notch1 signaling. Although not discussed in this research, crosstalk between these three pathways is also a pivotal factor for the fate of NSCs^52^. Our data provide novel insight into the molecular mechanisms of rEWST on NSCs and important preclinical references supporting the basis for further development of neurological function recovery promoted by NSCs recruitment.

## Material and Methods

### Cell culture

NE-4C (CRL-2925; ATCC, Manassas, VA) mouse NSCs were grown in Dulbecco’s minimal essential medium with high glucose (DMEM, Gibco), supplemented with 10% fetal bovine serum. Cells were cultured according to ATCC culture method guidelines and incubated at 37 °C, 5% CO_2_. NE-4C cells were dissociated with 0.05% trypsin-EDTA and resuspended in the incubator supplemented DMEM described above. The plated cells were then cultured in the incubator, adding DMEM at 37 °C and 5% CO_2_ for 24 h before treatment with 20 ng/mL Dkk-1 or DAPT cells, which incubated in designated treatments for 24 h before being lysed for collection. Cells from passage levels 4–5 were used in the present study. The cell culture medium was refreshed every 3 days.

### Cell proliferation test

To investigate the possible impact of rESWT on the viability/proliferation of NE-4C cells, different doses of rESWT (from 1.0 bar to 1.5 bar to 2.0 bar to 2.5 bar to 3.0 bar to 3.5 bar, 500 impulses, and 2 Hz) were used in the cultures. The proliferation of NE-4C cells was analyzed by MTT cell (Promega, Japan) proliferation assay. After being treated with rESWT, 5 × 10^3^ cells were seeded into 96-well plates to grow for 12, 24, 36, 48, 60, and 72 h. Then, 20 µl of MTT was added to each well, and culture plates were incubated at 37 °C for 2 h. The absorbance was measured by photometry at 490 nm. We chose 2.5 bar, 500 impulses, 2 Hz as a therapeutic dose.

### rESWT treatment

To evaluate the influence on cell differentiation *in vitro*, rESWT (STORZ MEDICAL AG, Switzerland) was applied to the cultures at a dose of 2.5 bar, 500 impulses, and 2 Hz, which maximized the therapeutic effects without significant reduction of the cell viability. Coupling was used to minimize the loss of shock wave energy at the interface between the transmitter and culture bottle. The surface of the culture bottle was evenly coated with the coupling agent, and the culture medium was filled with the culture solution. The transmitter of rESWT acted vertically on the upper part, and the processing time interval was 7 days. The control group was maintained under the same culture conditions, but without rESWT exposure.

### Immunofluorescence

Cells grown on coverslips were fixed with 4% paraformaldehyde, followed by treatment with 0.1 M glycine for 20 min at 25 °C and 0.1% Triton X-100 for additional 5 min at 25 °C to permeabilize. Cells were then incubated alternatively with the following primary antibodies: NSE (1:200, Abcam), β-tubulin III (1:1000, Abcam), Wnt3a (1:1000, Abcam), and β-catenin (1:200, Abcam). The primary antibodies were washed with PBS, and the cells incubated with goat anti-mouse IgG-FITC (1:50 in PBS; Cappel Research Products, Durham, NC, USA) and goat anti-rabbit IgG-Texas green (1:200; Jackson Immunoresearch Laboratories, West Grove, PA, USA) for 30 min at 25 °C. Nuclei were stained with DAPI (1:10,000 in PBS, Sigma Chemical Co., St. Louis, MO, USA). Coverslips were finally mounted with Mowiol in PBS for observation. Fluorescence signals were analyzed by conventional fluorescence or by scanning cells in series of 0.5-µm subsequent sections with an ApoTome System (Zeiss, Oberkochen, Germany) connected to an Axiovert 200 inverted microscope (Zeiss). Image analysis was then performed using Axiovision software (Zeiss). Observations were made in 10 microscopic fields randomly taken from three different experiments.

### Western Blot Analysis

Total protein was isolated with a Protein kit (Macherey-Nagel). Protein samples were separated on SDS-PAGE and transferred to nitrocellulose membranes. Protein bands were detected with mouse monoclonal primary antibodies against NSE (1:10000, Abcam), β-tubulin III (1:1000, Abcam), PI3K (1:1000, Abcam), AKT (1:2000, Abcam), Wnt3a (1:20000, Abcam), β-catenin (1:10000, Abcam), nestin (1:3000, Abcam), Notch1 (1:1000, Abcam), NICD (1:1000, Abcam), and Jagged1 (1:1000, Abcam) followed by goat anti-rabbit secondary antibody (1:10,000, Abcam, Cambridge, MA). The ECL Prime Western blotting detection reagent (GE Healthcare, Piscataway, NJ) was used for visualization with the Reliance 600 imaging system (Perkin Elmer, Waltham, MA). Blot images were captured and band intensities were quantified using GeneSnap and GeneTools (Perkin Elmer), respectively. Band intensities for proteins of interest were first normalized to GAPDH and then compared with the control group for each experiment.

### Real-time PCR Analysis

RNA was extracted using the Trizol reagent (Invitrogen, Carlsbad, CA) following the manufacturer’s protocol and eluted with 0.1% diethylpyrocarbonate (DEPC)-treated water. Then, 1 µg of total RNA was used for reverse transcription using a Takara synthesis kit (Takara) according to manufacturer’s instructions. The following primers were used: PI3K: 5′-CAAGGACGCTGGGAAATCTGT-3′ and 5′-AGGGGGCATCTCGTTGTCT-3′; AKT: 5′-CCTCACACTCCTCGCCCTAT-3′ and 5′-GCTTGGACACAAAGGCTGC-3′; NSE: 5′-GCATCTATGAGGCCCTGGAACTAA-3′ and 5′-TCAGTCCCATCCAGTTCCAACA-3′; β-tubulin III: 5′-GCGATGAGCACGGCATAGAC-3′ and 5′-GAAGGCACCACGCTGAAGGT-3′; Wnt3a: 5′-ATGAACCGCCACAACAAC-3′ and 5′-TTCTCCACCACCATCTCC-3′ β-catenin: 5′-GAACCCAGAAGGCACAGACA-3′ and 5′-GCGGGACACCTACTCTCATAC-3′; LRP-6: 5′-ACACTGGGACTGTCCTCTGCGA-3′ and 5′-CCTTTGGTCCTGGTTGCCCACTG-3′: Axin: 5′-CCCCCAACGCCATCTTCAA-3′ and 5′-CTGGGATTGCCCCGAGTG-3′; Frizzled: 5′-CGAGCGAGACCGCACCAACA-3′ and 5′-CGTTGCCCAGGTGCGAGATGTAG-3′; GSK3β: 5′-GGAAAGACAACAGACAAATCACCAT-3′ and 5′-ACCTCTTTGCTCTGCTCCTG-3′; Nestin: 5′-CAACCACAGGAGTGGGAACT-3′ and 5′-TCTGGCATTGACTGAGCAAC-3′; Notch1: 5′-CCGCTGTGAGTCGGTCATTA-3′ and 5′-GGCACCTACAGATGAATCCA-3′; Jagged1: 5′-TCCAGCCTCCAGCCAGTGAA-3′ and 5′-GGAAGGCTCACAGGCTATGT-3′; and Hes1: 5′-TTCAGCGAGTGCATGAACGA-3′ and 5′-GTAGGTCATGGCGTTGATCT-3′. Real-time PCR was performed using the iCycler Real-Time Detection System (iQ5 Bio-Rad) under optimized PCR conditions. The reaction was carried out in a 96-well plate using iQ SYBR Green Supermix (Bio-Rad) with the following conditions: 95 °C for 3 min, followed by 45 cycles at 95 °C for 10 s, and 60 °C for 30 s. The relative expression of the housekeeping gene (b-actin) was used for standardizing the reaction. Relative mRNA expression levels were calculated using the 2^−ΔΔCt^ method^53,54^. All assays included a negative control and were replicated three times. Results are reported as mean ± SD from three different experiments.

### Statistical analysis

Quantitative data are expressed as the mean SD. All analyses were conducted using SPSS statistical software. Statistical comparisons were performed by one-way ANOVA followed by the Least Significant Difference test to compare the differences at each time. A probability value < 0.05 was considered significant.

### Data Availability

All data generated or analysed during this study are included in this published article.
